# Risk of cancer associated with cardiac catheterization procedures during childhood: a cohort study in France

**DOI:** 10.1186/1471-2458-13-266

**Published:** 2013-03-22

**Authors:** Helene Baysson, Jean Luc Réhel, Younes Boudjemline, Jerôme Petit, Brigitte Girodon, Bernard Aubert, Dominique Laurier, Damien Bonnet, Marie-Odile Bernier

**Affiliations:** 1 Institut de Radioprotection et de Sûreté Nucléaire, PRP-HOM, SRBE, BP 17, Fontenay aux Roses, France; 2 Centre de Référence Malformations Cardiaques Congénitales Complexes, M3C, Hôpital Necker-Enfants malades, AP-HP, Université Paris Descartes, Sorbonne Paris Cité, Paris, France; 3 Centre de Référence Malformations Cardiaques Congénitales Complexes, M3C, Centre Chirurgical Marie Lannelongue, Le Plessis Robinson, France

**Keywords:** Interventional cardiology, Cancer risk, Children, Epidemiology, Cohort study, Radiation exposure

## Abstract

**Background:**

Radiation can be used effectively for diagnosis and medical treatment, but it can also cause cancers later on. Children with congenital heart disease frequently undergo cardiac catheterization procedures for diagnostic or treatment purposes. Despite the clear clinical benefit to the patient, the complexity of these procedures may result in high cumulative radiation exposure. Given children’s greater sensitivity to radiation and the longer life span during which radiation health effects can develop, an epidemiological cohort study is being launched in France to evaluate the risks of leukaemia and solid cancers in this specific population.

**Methods/design:**

The study population will include all children who have undergone at least one cardiac catheterization procedure since 2000 and were under 10 years old and permanent residents of France at the time of the procedure. Electronically stored patient records from the departments of paediatric cardiology of the French national network for complex congenital heart diseases (M3C) are being searched to identify the children to be included. The minimum dataset will comprise: identification of the subject (file number in the centre or department, full name, sex, date and place of birth), and characteristics of the intervention (date, underlying disease, type of procedure, technical details, such as fluoroscopy time and dose area product, (DAP), which are needed to reconstruct the doses received by each child). The cohort will be followed up through linkage with the two French paediatric cancer registries, which have recorded all cases of childhood leukaemia and solid cancers in France since 1990 and 2000, respectively. Radiation exposure will be estimated retrospectively for each child. 4500 children with catherizations between 2000 and 2011 have been already included in the cohort, and recruitment is ongoing at the national level. The study is expected to finally include a total of 8000 children.

**Discussion:**

This French cohort study is specifically designed to provide further knowledge about the potential cancer risks associated with paediatric cardiac catheterization procedures. It will also provide new information on typical dose levels associated with these procedures in France. Finally, it should help improve awareness of the importance of radiation protection in these procedures.

## Background

The main source of artificial radiation exposure in the general population is exposure for medical purposes [1]. In France, this accounts for 40% of the annual exposure of the entire population. Most of this exposure is related to diagnostic procedures, which are associated with low levels of ionising radiation. Despite recent technological innovations that reduce the doses delivered, the increased frequency of medical procedures has resulted in an increase in overall population exposure to ionising radiation, especially in the youngest and oldest age groups. A study conducted by the IRSN (Institute for Radiological Protection and Nuclear Safety) and the InVS (French Institute for Public Health Surveillance), based on health insurance records and a survey of a sample of hospitals, estimated that about 74.6 million diagnostic procedures using ionising radiation were performed in 2007 in France [2]. Moreover, recent years have seen an increase in the frequency of relatively high dose procedures, such as computed tomography (CT) scanning [3] and interventional procedures in cardiology [4-6].

Cardiac catheterization procedures (CCP) are medical interventions in which fluoroscopy is used to guide probes and guidewires into the circulatory system, to obtain hemodynamic and/or anatomical information for diagnostic purposes or to place a variety of devices, such as balloons, plugs and stents (Table 1) to correct or palliate various cardiac problems, for treatment purposes. The live birth incidence of congenital heart defects (CHD) is around 0.8% [7,8], and one sixth of the infants with CHD in developed countries will undergo CCP. With the technological progress, the role of the CCP has changed, moving from diagnostic to therapeutic. Therapeutic CCP have now become first line therapy for several specific CHD; they account for almost two thirds of CCP in expert centers. Although radiation doses during CCP are lower than in the past, procedures last longer as they have become more complex being mostly interventional [9].

**Table 1:** **Indications for diagnostic and therapeutic cardiac catheterization procedures in paediatrics (from **[5]**)**

	
Main indications for diagnostic cardiac catheterization procedures in pediatrics	
1	- Pulmonary arterial hypertension
2	- Pulmonary artery angiographies
3	- Evaluation of intracardiac shunts
Main indications for therapeutic cardiac catheterization procedures in pediatrics	
1	- Balloon dilatation (valves) - Valvuloplasty
2	- Balloon dilatation (vessels) – Angioplasty
3	- Patent Arterial Duct (PAD) closure
4	- Atrial Septal Defect (ASD) closure
5	- Ventricular Septal Defect (VSD) closure
6	- Embolisation of abnormal vessels
7	- Hybrid procedures

Several recent studies have investigated radiation doses in paediatric CCP [6,9-14]. These numbers remain limited in comparison with adult studies, probably due to the smaller number of centres performing these paediatric procedures [13]. Thus far, published doses for paediatric CCP, often available as effective doses (Table 2), range from 2.2 mSv to 12 mSv [14] for different types of standard paediatric CCP. But there is a wide variation in dose from one CCP to another and from one child to another (in particular, the child’s weight at the time of the procedure is a key factor to consider). The multiplicity of interventions in the same child can eventually lead to a non-negligible cumulative dose. Significant variation between paediatric cardiology units is also expected, as they use different machines and have operators of widely different levels of experience.

**Table 2:** Effective dose (mSv) for diagnostic and therapeutic paediatric CCP from the literature

**Study**	**Year**	**Diagnostic**				**Therapeutic**			
		**Min**	**Median**	**Mean**	**Max**	**Min**	**Median**	**Mean**	**Max**
Bacher	2005	0.6	4.6		23.2	1.0	6.0		37.0
Onnash	2007			3.6				5 *	
Yakoumakis	2009	0.16	2.90	3.71	16.44	0.38		5.00	25.01
El Sayed	2011			3.4				5.9	

(*) Range from 2.16 to 12.1, depending on the type of procedure.

### Epidemiological studies of cancer risks following diagnostic radiation exposure in children

Short-term effects of ionising radiation result primarily from irradiation at high doses. The severity of these effects is then directly proportional to the dose received; the effects are *deterministic*. For low doses, however, the effects are *stochastic*: they appear later, randomly, and their probability of occurrence increases with dose. Cancer is the most frequent of these stochastic effects [1]. It appears to be of greatest concern for children as several studies suggest that they are more susceptible than adults to the effects of ionising radiation: for a given dose, exposure during childhood is associated with a risk of cancer greater than if it had been received during adulthood [15]. Children also have more remaining years of life during which a radiation-induced cancer could develop. Cancers occur after a latency period, which evidence suggests is at least 5 to 10 years for most solid cancers and approximately 2 years for leukaemia.

Over the past 30 years, researchers have collected large quantities of epidemiological data about populations (all ages) exposed to ionising radiation for medical (therapeutic or diagnostic) purposes [16,17]. These studies are difficult to compare because they include populations of very different sizes (from a few hundred to several thousand patients), they are heterogeneous in age since some include children but also young adults, and their methodological quality varies widely [18]. The types of radiation considered also vary, as do the target organs and the exposure period — all factors that strongly affect dose.

Several cohort studies involving patients who received multiple diagnostic X-rays during childhood or adolescence to monitor tuberculosis or scoliosis have reported a risk of breast cancer that increased with dose and the number of radiographs [19-22]. It should be noted that the cumulative dose to the breast was relatively high, around 0.8 to 0.9 gray for tuberculosis follow-up [19,21] and 0.11 gray for monitoring scoliosis [20]. Case–control studies have found conflicting results, some showing an increased risk of cancer after exposure to radiological examinations in childhood [23-25], others finding no increased risk [26-28]. A first study analysing the risk of developing cancer after repeated CT examinations in the UK has just appeared [29]. The authors, assuming the typical doses used since 2001, suggest that 2-3 CT scans of the head could triple children’s risk of brain cancer and that 5-10 might triple their risk of leukaemia.

Only two cohort studies have assessed the association between the risk of cancer in children and radiation exposure during paediatric CCP. MacLaughlin studied 4891 Canadian children who had undergone at least one cardiac CCP before the age of 18 years between 1946 and 1968 and did not demonstrate a significant increase in leukaemia or in any other tumours in this population [30]. A second study, conducted by Modan, examined the records of 674 children who had undergone CCP for CHD between 1950 and 1970 and showed an excess number of solid cancers and of lymphomas [31]. Methodological limitations (no estimation of the doses received, types of CCP used unknown) might explain the inconsistency of these results. More recently, Ait-Ali applied risk models in the literature [32] and estimated a lifetime attributable risk of death from cancer equal to 1 in 1717 (0.06%) for boys (from 0 to 15 years) receiving an average of 7.1 mSv and equal to 1 in 859 (0.12%) in girls receiving an average of 9.4 mSv during CCP [33]. However, the available models, which are based mainly on Hiroshima and Nagasaki A-bomb survivors, have only limited information on the risk of cancer after exposure in early childhood [34].

In this context, our research project is the direct epidemiological follow-up of children who underwent CCP to evaluate their health status in terms of cancer and leukaemia.

## Methods/design

### Study aims

The objective of the study is to set up a cohort of children who underwent at least one CCP before the age of 10 years from 2000 through 2013 and assess their risk of solid cancer and leukaemia associated with radiation exposure from CCP during childhood. More specifically, the constitution of the cohort will allow us:

- To characterise the paediatric population that underwent CCP in France from 2000 through 2013;

- To obtain new information on typical dose levels for paediatric CCP in France during this period;

- To study the hypothesis of an excess risk of solid cancers and leukaemia attributable to ionising radiation exposure during CCP in children, by quantifying the dose response relation between radiation exposure and cancer risk and analysing potential modifying factors (age at first exposure, sex, etc.).

- To improve awareness of the importance of radiation protection during CCP in children.

### Type of protocol

A cohort study is being conducted. The principle of this type of protocol is the identification of a group of individuals about whom certain exposure information is collected (here children under 10 years old at the time of first CCP). The group is then followed forward in time to ascertain the occurrence of the diseases of interest (here solid cancer and leukaemia), so that for each individual, prior exposure information can be related to subsequent disease experience [35].

### Population and setting

**Figure 1:**
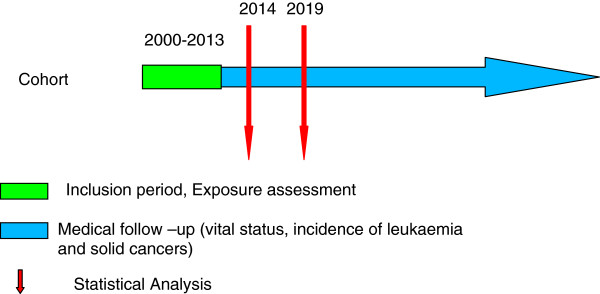
General overview of the study design.

The study population consists of patients who underwent at least one CCP (either for diagnostic or therapeutic purposes) before the age of 10 years and from 1 January, 2000, through 31 December, 2013 (Figure 1). Because of the lack of a national adult cancer registry, our study focuses on children exposed very young so that we can follow them in incidence data for at least 10 years, through the French paediatric cancer registries.

CCP in children are predominantly performed in tertiary hospitals in France. Fifteen centers have been identified, all members of the French national network for Complex Congenital Cardiac Defects-M3C, which treats about 80% of the children with CHD in France. In all, 4500 children recruited from the two centres coordinating the M3C-network (Necker Enfants malades, Paris, and Centre Chirurgical Marie Lannelongue, Le Plessis-Robinson) have already been included in the cohort. Recruitment is ongoing in these two centres and will be extended to other centres of the M3C network. The study is expected to finally include about 8000 children. This estimation is based on the average number of paediatric CCP performed in the two coordinating centers. These 8000 children, followed up for 15 years, should contribute about 120, 000 person-years. During the follow-up period, the expected number of solid cancers (from all causes) is 16 and the expected number of leukaemia cases 6 (Table 3). Thus, the cohort will have a statistical power of 80% to detect a SIR equal to 1.7 for solid cancers and 2.07 for leukaemia (with α = 0.05).

**Table 3:** **Smallest measurable SIRs for cancer and leukaemia for different scenarios (adapted from:**[36]**)**

			
Cohort size	8,000	25,000	32,000
Follow-up duration (years)	15	15	15
Person-years	120,000	375,000	480,000
Expected number of childhood cancer cases in the cohort^a^	16	51	65
Smallest measurable SIR (all cancers)^b^	1.7	1.4	1.3
Expected number of childhood leukaemia cases in the cohort^a^	5.6	17.6	22.6
Smallest measurable SIR (all leukaemia)^b^	2.1	1.6	1.5

a The annual standardised incidence rate of childhood cancer per 100,000 for the period 2000-2004 in France was 15.6 for all cancers and 4.6 for leukaemia (source: [37]).

b Smallest measurable SIR with α = 0.05 and β = 0.02.

### Data available for the cohort

Electronically stored patient records from the departments of paediatric cardiology of tertiary hospitals in France are being searched to identify the children to be included. The minimum dataset will comprise: identification of the patient (file number in the centre or service, full name, sex, date and place of birth and characteristics of the CCP (date of the procedure, age, weight, underlying disease, type of procedure, technical details including but not limited to fluoroscopy time and dose area product (DAP)).

### Follow-up of the children

Health status will be followed up by assessments in the future and every five years from 2014 (Figure 1). The vital status of each child will be identified by cross-linkage with the national vital status registry (Répertoire National d’Identification des Personnes Physiques (RNIPP)).

Up to age 15, follow-up of cancer incidence will be performed though cross linkage with the paediatric cancer registries (Registre des Tumeurs Solides de l’Enfant (RTSE) and Registre National des Hemopathies de l’Enfant (RNHE)), which since 1990 and 2000, respectively, have recorded all cases of childhood (i.e., under 15 years old at diagnosis) leukaemia and cancers in France. Above the age of 15 years, follow-up will be based on mortality status as France has no national cancer registry for adult cancers. Follow-up of morbidity through data from the medical insurance system (specifically, the SNII-RAM, an inter-health insurance scheme information system) may be available in the future.

### Dose assessment

Individual CCP-related doses will be assessed for each child included in the cohort (Figure 1). Exposure parameters (DAP, fluoroscopy time) will be retrieved from the dose-recording system. The DAP, which represents the dose in air measured at a given distance from the X-ray tube multiplied by the area of the x-ray at that distance [33], will be used as a surrogate for radiation exposure [9]. Organ doses, especially to the lung, the oesophagus, and the thyroid will then be calculated with PCXMC software [38]. After the development of several assumptions, this programme, based on the Monte Carlo method and developed to calculate patients’ organ doses in medical examinations, could be used for the simulation of CCP [39].

The proposed study does not consider radiation exposure outside the participating centres. However, it is planned to address the children’s exposure to CT scans, which account for a major contribution to their medical exposure dose. We will be able to cross-match our data with data from the childhood French CT-scan cohort (“Cohort Enfant Scanner”) set up by the IRSN [3] for the children included in both cohorts. For children who underwent CCP in centres not participating in the “Enfant Scanner” scanner, it will be necessary to go back to medical files to obtain information about the CT scans performed.

### Planned analysis

A detailed analysis plan was prepared as part of the protocol. The statistical analyses will comprise:

A description of the paediatric population that underwent CCP in France from 2000 through 2013 (age, disease, etc.);

Description of the doses from paediatric CCP;

A comparison of the incidence of childhood cancers and leukaemia in the cohort with that of the French paediatric population.

Depending on the statistical power available, it should be possible to quantify the dose–response relation between the irradiation received during CCP and the occurrence of cancer or leukaemia in childhood or adulthood. Modifying factors, such as age at exposure and sex, will be also considered in the risk modelling.

### Ethical aspects

The French national data protection authority (Commission Nationale Informatique et Liberté (CNIL)) (n° 911112 of December 12, 2011) has approved the study and the conditions of storage of personal data and use of anonymised dosimetric and clinical data.

### Associated teams and partnership added value

The study will be conducted by the Laboratory of Epidemiology of the Institute of Radioprotection and Nuclear Safety (IRSN) in close collaboration with IRSN’s Medical Expertise Unit (UEM) for dosimetric expertise and with the paediatric cardiology units participating in this study. The follow-up of the cohort for incidence of solid cancers and leukaemia will be based on collaboration with the national registries of paediatric cancers (RTSE and RNHE).

Collaboration is also planned with researchers from the University of Newcastle (UK), where Dr M Pearce’s group is conducting a similar study.

The complementary expertise of the three units working together — in paediatric cardiology (Reference Centre for Complex Congenital Cardiac Defects –M3C, Hôpital Necker), medical dosimetric expertise (Unit of Medical Expertise of IRSN), and epidemiology in the field of ionising radiation (Laboratory of Epidemiology of IRSN) — is a major strength of this study.

This research will also benefit from the experience developed in the *Cohort Enfant Scanner* study, also conducted by the IRSN Laboratory of Epidemiology. This national cohort now includes nearly 90 000 children [3]. The French data are integrated in the European EPI-CT project, which is coordinated by the International Agency for Research on Cancer (IARC), (ref. http://epi-ct.iarc.fr/).

### Time plan

Initial inclusion data collection began in October 2011 and will continue through 2013. Thus, as early as 2014, the statistical analysis will provide the first answers about characteristics of the paediatric population undergoing CCP in France, of doses from paediatric CCP, and on the incidence of cancers in this population. Subsequent analyses of cancer risk will be conducted periodically.

## Discussion

CCP play a crucial role in the diagnosis and treatment of congenital heart diseases. The justification of these procedures is clear: they make it possible to avoid complicated invasive surgery. These procedures are, however, among the radiological procedures with the highest patient radiation dose. In the study by Ait-Ali, conventional x-ray examinations accounted for 5% of the collective effective dose and three types of procedures were responsible for the remaining 95%: diagnostic CCP, therapeutic CCP and CT scans [33]. Several authors have discussed the long-term effects, such as cancer, following diagnostic radiation exposure in children [16-18]. However, information on cancer risks associated with CCP during early childhood nonetheless remains limited, and authors have stressed the utility of setting up epidemiological studies on the cancer risks associated with radiation exposure during CCP [40]. Such a study is more feasible today than in the past, for the long-term outcome of the underlying cardiac diseases has improved greatly in the past decade, and now excellent long-term survival is the rule, rather than the exception [33]. Adult survivors of surgically repaired CHD are a large and growing population, estimated to be one million in the US in the year 2000, compared with an estimated 300 000 in 1980 [33].

In this context, our objective is to set up a cohort of children who have undergone at least one CCP before the age of 10 years to assess the risk of solid cancers and leukaemia associated with radiation exposure. The inclusion of the children is currently ongoing *via* the French national network for Complex Congenital Cardiac defects-M3C. As most CCP are performed during the first year of life, our cohort will include very young children. Focusing our study on young children will also allow us to follow them in incidence data for at least 10 years, through the French paediatric cancer registries. Nevertheless, due to the medical context, this population of children is characterised by very good medical follow-up with regular medical visits. Specifically, details regarding the child’s health at the time of the CCP will be reliably available from the French hospital discharge summary database (*programme de médicalisation des systèmes d’information,* PMSI), based upon diagnosis-related groups (DRG) [41]. This will make it possible to take into account such confounding factors as Down Syndrome, which is known to be associated with both cancer and heart defects.

The previous absence of any such cohort in France highlights the novelty of the project at the national level. A similar study is currently being conducted in the UK by Dr M. Pearce, University of Newcastle, and the feasibility of a pooled analysis to increase the statistical power of these individual studies is under discussion. Even with complete and accurate follow-up, it must be acknowledged that our study by itself will have limited statistical power. The size of the French cohort was estimated based on the average number of CCP performed in the two main centres (Necker Enfants malades and Centre Chirurgical Marie Lannelongue). As indicated in Table 3, this sample size will be sufficient to detect an overall excess risk of 1.7 for all cancer incidence, but smaller levels are expected. Pooling data from France with data from UK will allow detecting small excess risk of 1.3.

A key aspect of the proposed study will be the assessment of individual doses received by children during CCP. In general, radiation doses increase with age and procedural complexity [42]. But acquisition data, tube angulation, total fluoroscopy time and total cinematography time depend strongly on the complexity of the CHD, the patient’s size and morphology, and on the physician’s technical skills and experience [39]. Both of the major participating paediatric cardiology departments (Hospital Necker and Chirurgical Centre Marie Lannelongue) have highly stable staff, which reduces the variability around the dose due to an operator effect.

However, there is a wide variation in the doses between different CCP as there is no standard protocol for this procedure, which is actually many different types of procedures. Moreover, paediatric cardiac patients are a relatively inhomogeneous group in the sense that there are many different types of CHD in children that require different types of procedures. Consequently, the radiologic interventions do not generally follow a standardised scheme. Furthermore, it is a relatively young and rapidly evolving field. Although some groups share similar nomenclature systems or coding lists, the field has not yet adopted a comprehensive and universally accepted nomenclature system for CCP [43]. Nevertheless, a common language, or system of nomenclature, is imperative as the study will be carried out in several centres in France. Principally, two broad categories will be defined: diagnostic CCP and therapeutic CCP. Subcategories for therapeutic CCP have been identified according to their main indications in children: valvuloplasty, angioplasty, patent arterial duct (PAD) closure, atrial septal defect (ASD) closure, and ventricular septal defect (VSD) closure.

The estimation of individual doses is also complicated by the fact that the image acquisition parameters for calculating the dose level of a given body are rarely available. In our study, the magnitudes recorded automatically in recent years are the fluoroscopy time and the DAP (Dose Area Product). The effective dose can be calculated from these quantities and can be used to compare between different procedures. Nevertheless, the effective dose is a quantity calculated from tissue weighting factors unrelated to sex and age, defined by the International Commission on Radiological Protection [44], and it does not take into account the increased radiosensitivity of children compared to adults. To obtain more comprehensive information on children’s exposure during CCP, our research project will calculate organ doses, including to the lungs, oesophagus, and thyroid, with appropriate software (PCXMC) [38].

By addressing the exposures received during childhood, our research will provide some answers about individual variability in cancer risk, particularly, about the effect of age at exposure. Other large-scale studies on the effects of medical exposure in childhood have been developed in France over the past five years. The European project EPI-CT (Epidemiological study to quantify risks for paediatric computerised tomography and to optimise doses) study, currently underway at the international level, is designed to gather data from nine national cohorts of children undergoing one or more CT scans to determine whether there is an excess of cancer cases attributable to CT exposure during childhood [3,29,36]. Another study examining the health effects of medical exposure during childhood is ELFE (Etude Longitudinale Française depuis l’Enfance), launched in April 2011 in France [45], intended to follow 20,000 children from birth to adulthood and analyse the impact of various factors (social, environmental, nutritional, etc.) on their physical, psychological and social development. Medical exposure to ionising radiation is one of these factors and will be collected prospectively through a questionnaire specifying the type of examination (radiography, CT scans, etc.), the number, and the anatomical zone explored.

## Conclusion

Technological advances have made it possible for CCP to play a therapeutic as well as a diagnostic role. In various CHD, it can allow surgical procedures to be postponed or even replaced. But CCP can also involve potentially non-negligible doses of radiation to the patients. This issue is particularly relevant for children as they are relatively more sensitive to ionising radiation than adults are and have a longer mean lifetime expectancy. Our cohort study is specifically designed to provide further knowledge on the potential cancer risk associated with paediatric CCP. This nationwide study will provide comprehensive information on typical levels of doses for paediatric interventional cardiology procedures in France. In the meantime, it is very important to optimise procedures and keep doses to paediatric patients as low as possible.

## Competing interests

The authors declare that they have no competing interests.

## Authors’ contribution

HB, JLR, DB, YB, JP, and MOB were responsible for identifying the research question, and contributing to drafting of the study protocol. BG, BA and DL have contributed to the development of the protocol and study design, as members of the research team. HB, JLR and MOB were responsible for the drafting of this paper, although all authors read and approve the manuscript.

## Funding

This work is supported by a public source of funding from the Institut National du Cancer (INCa), the French National Cancer Institute, grant number INCa_6139.

## Pre-publication history

The pre-publication history for this paper can be accessed here:

http://www.biomedcentral.com/1471-2458/13/266/prepub
